# Machine learning to predict early recurrence after oesophageal cancer surgery

**DOI:** 10.1002/bjs.11461

**Published:** 2020-01-30

**Authors:** S. A. Rahman, R. C. Walker, M. A. Lloyd, B. L. Grace, G. I. van Boxel, B. F. Kingma, J. P. Ruurda, R. van Hillegersberg, S. Harris, S. Parsons, S. Mercer, E. A. Griffiths, J. R. O'Neill, R. Turkington, R. C. Fitzgerald, T. J. Underwood, the OCCAMS Consortium, Ayesha Noorani, Rachael Fels Elliott, Paul A.W. Edwards, Nicola Grehan, Barbara Nutzinger, Jason Crawte, Hamza Chettouh, Gianmarco Contino, Xiaodun Li, Eleanor Gregson, Sebastian Zeki, Rachel de la Rue, Shalini Malhotra, Simon Tavaré, Andy G. Lynch, Mike L. Smith, Jim Davies, Charles Crichton, Nick Carroll, Peter Safranek, Andrew Hindmarsh, Vijayendran Sujendran, Stephen J. Hayes, Yeng Ang, Shaun R. Preston, Sarah Oakes, Izhar Bagwan, Vicki Save, Richard J.E. Skipworth, Ted R. Hupp, J. Robert O'Neill, Olga Tucker, Andrew Beggs, Philippe Taniere, Sonia Puig, Timothy J. Underwood, Fergus Noble, James P. Byrne, Jamie J. Kelly, Jack Owsley, Hugh Barr, Neil Shepherd, Oliver Old, Jesper Lagergren, James Gossage, Andrew Davies Fuju Chang, Janine Zylstra, Vicky Goh, Francesca D. Ciccarelli, Grant Sanders, Richard Berrisford, Catherine Harden, David Bunting, Mike Lewis, Ed Cheong, Bhaskar Kumar, Simon L. Parsons, Irshad Soomro, Philip Kaye, John Saunders, Laurence Lovat, Rehan Haidry, Victor Eneh, Laszlo Igali, Michael Scott, Shamila Sothi, Sari Suortamo, Suzy Lishman, George B. Hanna, Christopher J. Peters, Anna Grabowska

**Affiliations:** ^1^ Cancer Sciences Unit University of Southampton Southampton UK; ^2^ Department of Public Health Sciences and Medical Statistics University of Southampton Southampton UK; ^3^ Department of Surgery Nottingham University Hospitals NHS Trust Nottingham UK; ^4^ Department of Surgery Portsmouth Hospitals NHS Trust Portsmouth UK; ^5^ Department of Upper Gastrointestinal Surgery University Hospitals Birmingham NHS Foundation Trust Birmingham UK; ^6^ Cambridge Oesophagogastric Centre Addenbrookes Hospital, Cambridge University Hospitals Foundation Trust Cambridge UK; ^7^ Hutchison/Medical Research Council Cancer Unit University of Cambridge Cambridge UK; ^8^ Centre for Cancer Research and Cell Biology Queen's University Belfast Belfast UK; ^9^ Department of Surgery University Medical Centre Utrecht the Netherlands

## Abstract

**Background:**

Early cancer recurrence after oesophagectomy is a common problem, with an incidence of 20–30 per cent despite the widespread use of neoadjuvant treatment. Quantification of this risk is difficult and existing models perform poorly. This study aimed to develop a predictive model for early recurrence after surgery for oesophageal adenocarcinoma using a large multinational cohort and machine learning approaches.

**Methods:**

Consecutive patients who underwent oesophagectomy for adenocarcinoma and had neoadjuvant treatment in one Dutch and six UK oesophagogastric units were analysed. Using clinical characteristics and postoperative histopathology, models were generated using elastic net regression (ELR) and the machine learning methods random forest (RF) and extreme gradient boosting (XGB). Finally, a combined (ensemble) model of these was generated. The relative importance of factors to outcome was calculated as a percentage contribution to the model.

**Results:**

A total of 812 patients were included. The recurrence rate at less than 1 year was 29·1 per cent. All of the models demonstrated good discrimination. Internally validated areas under the receiver operating characteristic (ROC) curve (AUCs) were similar, with the ensemble model performing best (AUC 0·791 for ELR, 0·801 for RF, 0·804 for XGB, 0·805 for ensemble). Performance was similar when internal–external validation was used (validation across sites, AUC 0·804 for ensemble). In the final model, the most important variables were number of positive lymph nodes (25·7 per cent) and lymphovascular invasion (16·9 per cent).

**Conclusion:**

The model derived using machine learning approaches and an international data set provided excellent performance in quantifying the risk of early recurrence after surgery, and will be useful in prognostication for clinicians and patients.

## Graphical abstract

Early recurrence after surgery for adenocarcinoma of the oesophagus is common. A risk prediction model was derived using modern machine learning methods that accurately predicts risk of early recurrence using postoperative pathology.

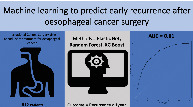

Machine learning may help

## Abstract (es)

**Antecedentes:**

la recidiva precoz del cáncer tras esofaguectomía es un problema frecuente con una incidencia del 20‐30% a pesar del uso generalizado del tratamiento neoadyuvante. La cuantificación de este riesgo es difícil y los modelos actuales funcionan mal. Este estudio se propuso desarrollar un modelo predictivo para la recidiva precoz después de la cirugía para el adenocarcinoma de esófago utilizando una gran cohorte multinacional y enfoques con aprendizaje automático.

**Métodos:**

Se analizaron pacientes consecutivos sometidos a esofaguectomía por adenocarcinoma y que recibieron tratamiento neoadyuvante en 6 unidades de cirugía esofagogástrica del Reino Unido y 1 de los Países Bajos. Con la utilización de características clínicas y la histopatología postoperatoria se generaron modelos mediante regresión de red elástica (*elastic net regression*, ELR) y métodos de aprendizaje automático Random Forest (RF) y XG boost (XGB). Finalmente, se generó un modelo combinado (Ensemble) de dichos métodos. La importancia relativa de los factores respecto al resultado se calculó como porcentaje de contribución al modelo.

**Resultados:**

En total se incluyeron 812 pacientes. La tasa de recidiva a menos de 1 año fue del 29,1%. Todos los modelos demostraron una buena discriminación. Las áreas bajo la curva ROC (AUC) validadas internamente fueron similares, con el modelo Ensemble funcionando mejor (ELR = 0,791, RF = 0,801, XGB = 0,804, Ensemble = 0,805). El rendimiento fue similar cuando se utilizaba validación interna‐externa (validación entre centros, Ensemble AUC = 0,804). En el modelo final, las variables más importantes fueron el número de ganglios linfáticos positivos (25,7%) y la invasión linfovascular (16,9%).

**Conclusión:**

El modelo derivado con la utilización de aproximaciones con aprendizaje automático y un conjunto de datos internacional proporcionó un rendimiento excelente para cuantificar el riesgo de recidiva precoz tras la cirugía y será útil para clínicos y pacientes a la hora de establecer un pronóstico.

## Introduction

Oesophageal adenocarcinoma carries a poor prognosis. Among the less than 40 per cent of patients who are candidates for curative treatment^1^, the 5‐year survival rate remains approximately 25–50 per cent in randomized trials^2^, ^3^, ^4^ and rarely exceeds 50 per cent in case series.

Early recurrence (less than 1 year) after surgery is a feared outcome, with rates of 20–30 per cent frequently reported^3^, ^4^, ^5^, despite the increasing uptake of neoadjuvant chemotherapy (NACT) and neoadjuvant chemoradiotherapy (NACRT). This is of particular concern because recovery from oesophagectomy is often long and the risk of major complications (Clavien–Dindo grade III–V) is as high as 30 per cent^6^. Many patients have not recovered from the primary cancer treatment when they experience recurrence.

In an ideal setting, prediction of early recurrence before embarking on a multimodal surgical pathway would provide useful information for patients and clinicians. However, staging information correlates poorly between preoperative and postoperative settings^7^, and genomic information is not yet able to predict outcome. Even the most robust preoperative models for prediction have a modest performance at best^8^. In contrast, postoperative information, although not able to influence surgical treatment decisions, is more prognostic and potentially informative for patients. It may also be helpful in making decisions on the merits of adjuvant therapy, further refining the high‐risk group of patients in whom novel adjuvant treatments are currently being considered.

Naive logistic regression has been the dominant approach to binary outcome prediction in clinical medicine for decades. Adoption of modern modified regression and machine learning techniques has been limited, in part owing to concerns over computational complexity and reliability. However, an increasing body of evidence has demonstrated that they outperform traditional techniques in predictive performance^9^, ^10^, although this is debatable^11^. In part, the appeal of these approaches lies in their ability to model complex non‐linear relationships that are common in cancer data, and which are challenging to model effectively with logistic/linear approaches. The increasing accessibility of software design now also allows the relatively straightforward deployment of these black‐box techniques.

The Oesophageal Cancer Clinical and Molecular Stratification (OCCAMS) Consortium^12^ previously published a multicentre UK cohort study that assessed survival according to Mandard Tumour Regression Grade (TRG)^13^. This study included patients who had undergone oesophagectomy for adenocarcinoma of the oesophagus or gastro‐oesophageal junction (GOJ) preceded by NACT. A clinically meaningful response to NACT was limited to TRG 1–2 only, which represented approximately 15 per cent of patients. The present study used this database, supplemented with an international cohort from the Netherlands, and machine learning techniques to develop and validate a clinically useful predictive model for early recurrence in oesophageal adenocarcinoma.

## Methods

The OCCAMS Consortium is a UK‐wide multicentre consortium set up to facilitate clinical and molecular stratification of oesophagogastric cancer. It has ethical approval for biological sample collection and analysis in conjunction with detailed clinical annotation (Research Ethics Committee number 10/H0305/1). Data collection and participation in research were approved by institutional ethics committees at each OCCAMS site and University Medical Centre (UMC) Utrecht.

### Source of data

Data were sourced from six tertiary oesophagogastric centres in the UK, as described previously^12^. Briefly, the records of consecutive patients from each centre who underwent a planned curative oesophagectomy for adenocarcinoma between 2000 and 2013, and also received NACT (platinum‐based triplet or cisplatin and 5‐fluorouracil) were reviewed and collated. Treatment was decided by a multidisciplinary team at individual institutions. Neoadjuvant treatment was considered for patients with locally advanced (cT2+) or node‐positive disease according to local and national guidelines. Clinical, pathological, recurrence and survival data were recorded. Data from one of the original centres were incomplete to the extent that modelling could not take place and were excluded *a priori*. To include NACRT as a factor in the model, further patients were identified from University Hospitals Southampton and UMC Utrecht, where CROSS (Chemoradiation for Oesophageal Cancer Followed by Surgery Study)‐type NACRT^4^ has been the standard of care for oesophageal adenocarcinoma for a number of years. Patients whose tumours were deemed unresectable at the time of surgery or who had metastatic disease on postoperative histology (pM1) were excluded from the analysis.

The primary outcome measure was early recurrence, defined as confirmed local, regional or distant recurrence at less than 1 year from the date of surgery^5^, ^8^, ^14^. Missing data were treated as being missing completely at random and handled by listwise deletion. Modelling was based on a complete‐case analysis.

### Predictor characteristics

Univariable statistics were calculated using non‐parametric Mann–Whitney *U* and χ^2^ tests. The predictive models were generated on the whole data set. All available variables were included in the analysis. A circumferential resection margin (CRM) of less than 1 mm was considered to be involved (and hence R1), in accordance with Royal College of Pathologists (RCP) guidelines^15^. Tumour grade and TRG^13^ were assessed by dedicated gastrointestinal histopathologists who were blinded to the clinical data. TRG was used to distinguish between responders (TRG 1–2) and non‐responders (TRG 3–5), in line with the previous publication based on this data set^12^. To increase the yield of information from lymph node data, both the number of positive lymph nodes and total lymph node harvest were considered as absolute numbers. For the regression model, linearity was assumed for continuous variables. The variables used to predict outcome were: age, sex, tumour location, type of neoadjuvant therapy, response to neoadjuvant therapy (TRG), ypT category, lymphovascular invasion, completeness of resection, grade of differentiation, number of positive lymph nodes and total number of lymph nodes examined.

### Model building and validation

Elastic net regularized logistic regression (ELR)^16^ was used along with two machine learning techniques: random forest (RF)^17^ and extreme gradient boosting (XG boost, XGB)^18^. ELR applies a combination of the ridge and lasso penalties^19^, ^20^ with the benefits of both (partly minimization of overfitting and variable selection). RF combines a specified number of decision trees (typically around 1000) created on random subsets of the data set, and is probably the most widely used machine learning approach in the medical literature. XGB attempts to improve sequentially by generating models to explain where the original model fails and then repeating this process (typically around 1000 times), while simultaneously applying regularization to minimize overfitting. Having generated individual models, these were combined to generate overall predictions^21^, an approach that theoretically is particularly beneficial when using diverse model types (such as those described above) that capture different elements of patients' risk profiles.

For ELR, the optimal α and λ hyperparameters (penalty severities) were selected by grid search using tenfold cross‐validation with five repeats during model generation and log loss as the metric for optimization. The RF model was derived from 1000 decision trees and hyperparameter tuning was conducted in a similar fashion (for number of variables per tree, split rule and minimum node size). The XGB model was again derived by cross‐validation of hyperparameters (number of optimization rounds, maximum tree depth, minimum weight in each child node, minimum loss reduction (γ), regularization penalty (η) and subsampling for regularization). Full details of hyperparameter tuning are available in *Appendix* *S2* (supporting information). These three models were then combined to generate the final (ensemble) model by generating a linear blend of predicted probabilities using logistic regression.

Discrimination of the models was assessed using the area under the receiver operator characteristic (ROC) curve (AUC). In the context of this paper, if two random patients were selected, one with recurrence of cancer at less than 1 year and one disease‐free at 1 year, the AUC is equivalent to the probability the model will score the patient with recurrence higher than the patient without. Internal validation was performed using 0·632 bootstrapping, with 1000 resampled data sets. Bootstrapping was preferred for internal validation over splitting the cohort into derivation and validation sets, as this has been shown to reduce bias and improve overall model performance, particularly with moderately sized data sets^22^, ^23^, ^24^. Calibration was assessed visually and formally with the Hosmer–Lemeshow test. As the data set contains multiple centres with small numbers of patients, an internal–external validation procedure was opted for, as advocated by Steyerberg and Harrell^25^. This entails generating models on all centres apart from one and validating the model on the remaining centre. This process is then repeated leaving each centre out sequentially, and a mean calculated. This method demonstrates how the model performs in external data while also allowing the whole data set to be used for training.

Unadjusted tree models (such as RF, which is included in the ensemble model) and other maximum margin methods typically calibrate poorly as a consequence of their methodology, with predicted probabilities biased towards the centre. To allow meaningful interpretation of probability, isotonic regression was used to scale probabilities on the final model, as described previously^26^, ^27^.

In contrast to logistic regression, assessing global variable importance is challenging using machine learning techniques and to an extent they are black boxes. As coefficients, as seen in logistic regression, are not used, an alternative method is required. The VarImp function of the caret R package was used, where ROC curves are generated for the outcome for each individual predictor, and the contribution to the global ROC curve calculated as a percentage. Owing to the nature of higher‐order interactions present in the model, variable importance in individual predictions must be calculated independently. The mean marginal contribution of each variable was calculated (change from the mean prediction; Shapley value^28^) for individual predictions. A similar approach was used by Nanayakkara and colleagues^29^ for analysing in‐hospital mortality following cardiac arrest.

Data analysis was conducted using R version 3.5.3 (R Foundation for Statistical Computing, Vienna, Austria). Models were trained using the caret^30^ and caretEnsemble^31^ packages. Individual variable importance was calculated using iml^32^. All are available at https://CRAN.R‐project.org/. The full R code to train the models is available in *Appendix* *S2* (supporting information), along with a list of packages used.

The calibrated final model was designed using R Shiny^33^ (available freely at https://uoscancer.shinyapps.io/EROC/). No data entered into the model were collected or stored.

## Results

**Figure 1:**
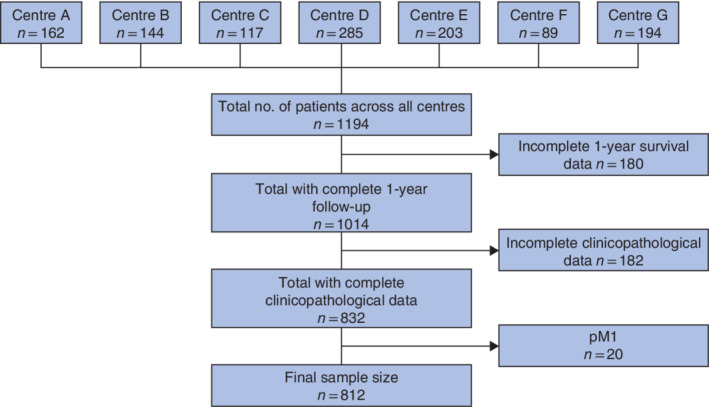
Study flow diagram

A total of 812 patients from seven centres were included in model training (*Fig*. 1). Median age was 64 years and most patients were men (84·6 per cent). The majority of tumours were at the GOJ (55·5 per cent), and there were high proportions of locally advanced tumours (66·7 per cent ypT3–4) and node‐positive disease (61·0 per cent). First recurrence of cancer within 1 year of surgery was identified in 236 patients (29·1 per cent). Patients in the early recurrence group were significantly less likely to have responded to neoadjuvant treatment (8·5 *versus* 21·7 per cent), had worse ypT and ypN categories, R1 resection rate and grade of differentiation, and were more likely to have lymphovascular invasion (all *P* < 0·001) (*Table* 1). Clinicopathological data are summarized by centre and type of adjuvant therapy in *Tables* *S1* and *S2* respectively (supporting information).

**Table 1:** Clinicopathological data for whole cohort and according to early recurrence

	All patients (*n* = 812)	No early recurrence (*n* = 576)	Early recurrence (*n* = 236)	*P* †
**Age (years)** *	64·0 (28–83)	63·9 (28–81)	64·1 (38–83)	0·855§
**Sex ratio (M** : **F)**	687 : 125	487 : 89	200 : 36	0·944
**Tumour site**				0·352
Oesophagus	361 (44·5)	250 (43·4)	111 (47·0)	
GOJ	451 (55·5)	326 (56·6)	125 (53·0)	
**Tumour Regression Grade**				< 0·001
TRG 1–2	145 (17·9)	125 (21·7)	20 (8·5)	
TRG 3–5	667 (82·1)	451 (78·3)	216 (91·5)	
**ypT category**				< 0·001
ypT0	33 (4·1)	28 (4·9)	5 (2·1)	
ypT1	96 (11·8)	87 (15·1)	9 (3·8)	
ypT2	141 (17·4)	125 (21·7)	16 (6·8)	
ypT3	495 (61·0)	320 (55·6)	175 (74·2)	
ypT4	47 (5·8)	16 (2·8)	31 (13·1)	
**No. of positive LNs** *	1 (0–41)	0·5 (0–30)	4 (0–41)	< 0·001§
**ypN > 0**	495 (61·0)	288 (50·0)	207 (87·7)	< 0·001
**Total no. of lymph nodes** *	24 (0–75)	24 (0–75)	23 (6–61)	0·805§
> 15	688 (84·7)	481 (83·5)	207 (87·7)	0·134
**Lymphovascular invasion**	372 (45·8)	202 (35·1)	170 (72·0)	< 0·001
**R1 resection**	231 (28·4)	118 (20·5)	113 (47·9)	< 0·001
**Tumour grade (differentiation)**				< 0·001
Well	63 (7·8)	55 (9·5)	8 (3·4)	
Moderate	300 (36·9)	233 (40·5)	67 (28·4)	
Poor/anaplastic	449 (55·3)	288 (50·8)	161 (68·2)	
**Neoadjuvant treatment**				0·061
NACT	657 (80·9)	476 (82·6)	181 (76·7)	
NACRT	155 (19·1)	100 (17·4)	55 (23·3)	

Values in parentheses are percentages unless indicated otherwise;

* values are median (range). GOJ, gastro‐oesophageal junction; LN, lymph node; NACT, neoadjuvant chemotherapy; NACRT, neoadjuvant chemoradiotherapy.

† χ^2^ test, except

^‡^Mann–Whitney *U* test.

### Model performance: discrimination

**Figure 2:**
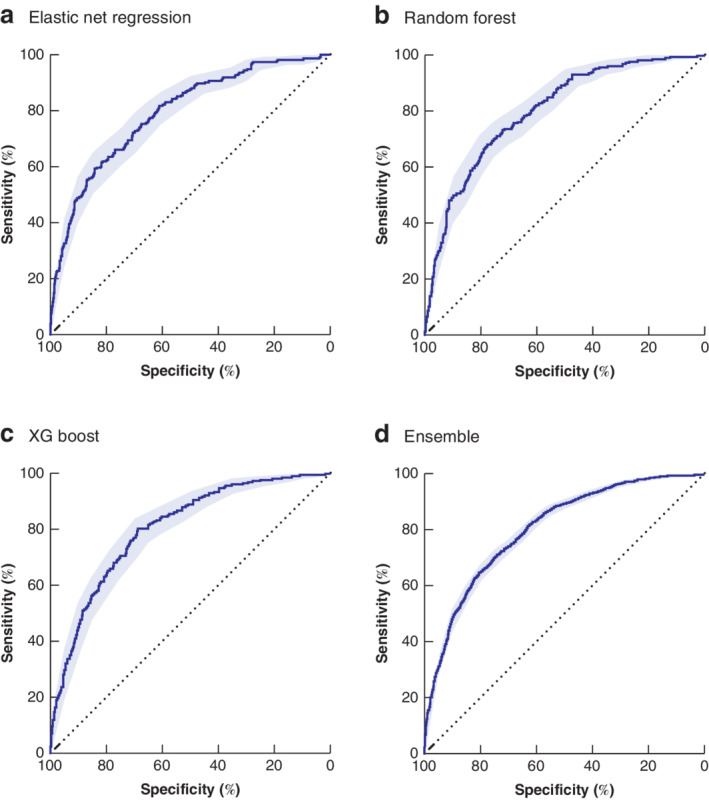
Model discrimination via 0·632 bootstrap Receiver operating characteristic (ROC) curves for **a** elastic net regression (area under the curve (AUC) 0·791, 95 per cent c.i. 0·757 to 0·826), **b** random forest (AUC 0·801, 0·769 to 0·834), **c** XG boost (AUC 0·804, 0·772 to 0·836) and **d** ensemble (AUC 0·805, 0·790 to 0·819). The shaded area represents the 95 per cent confidence interval.

Discrimination was assessed in the training set, internally (via bootstrapping) and internally–externally (across centres). All models demonstrated excellent discrimination on the training set (apparent discrimination). The RF model performed the best (AUC 0·980), followed by the ensemble model (0·902), XGB (0·849) and ELR (0·805) (*Table* 2). On internal validation, the ensemble model had the best performance (AUC 0·805) and the ELR the worst (0·791) (*Fig*. 2). Individual centre internal‐external validation ROC curves are available in *Fig*. *S3* (supporting information).

**Table 2:** Model discrimination

	Area under the curve		
	Apparent	Internal validation	Internal–external validation
Elastic net regression	0·805 (0·772, 0·838)	0·791 (0·757, 0·826)	0·798 (0·713, 0·883)
Random forest	0·980 (0·972, 0·987)	0·801 (0·769, 0·834)	0·805 (0·721, 0·889)
XG boost	0·849 (0·822, 0·877)	0·804 (0·772, 0·836)	0·800 (0·716, 0·883)
Ensemble	0·902 (0·881, 0·992)	0·805 (0·790, 0·819)	0·804 (0·721, 0·887)

Values in parentheses are 95 per cent confidence intervals.

### Model performance: calibration

**Figure 3:**
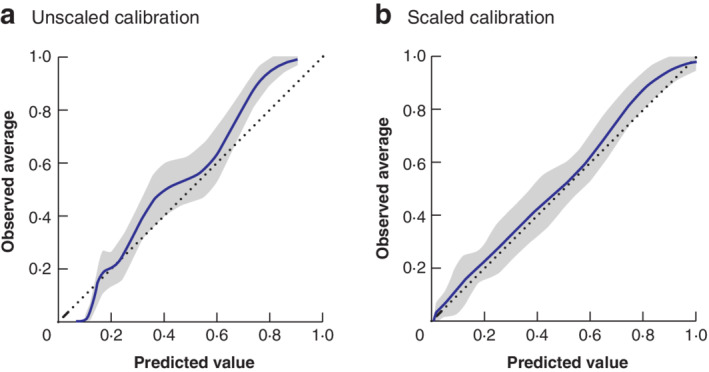
Ensemble model calibration before and after adjustment **a** Unscaled calibration (intercept 0·395, slope 1·574) and **b** scaled calibration (intercept 0·143, slope 0·988). The shaded area represents two standard errors.

Calibration on the training set was visually best in the ELR, and worst in the RF and ensemble models (*Fig*. *S1*, supporting information). This was corroborated by the Hosmer Lemeshow test (*P* = 0·806 for ELR, *P* < 0·001 for RF, *P* = 0·030 for XGB, *P* < 0·001 for ensemble). Probabilities generated by the final model were scaled using isotonic regression. Calibration before and after scaling is shown in *Fig*. 3. A calibration table can be found in *Table* *S3* (supporting information). The Hosmer–Lemeshow test gave a χ^2^ value of 38·0 (*P* < 0·001) before and 4·5 (*P =* 0·806) after scaling. Similarly, the Brier score, a measure of overall model performance, also improved from 0·119 to 0·114.

### Variable importance

Coefficients and odds ratios cannot be generated for these models. Therefore, variable importance as a percentage contribution to the model was computed (*Table* 3). Overall, the most influential predictor variable was number of positive lymph nodes (25·7 per cent), followed by lymphovascular invasion (16·9 per cent). There was considerable variability in importance across models. For example, age contributed 0·3 per cent to the ELR model, 18·2 per cent to the RF model, 10·2 per cent to the XGB model and 9·6 per cent to the final model.

**Table 3:** Variable importance

	Importance (%)			
	Elastic net regression	Random forest	XG boost	Ensemble (final model)
Age	0·3	18·2	10·2	9·6
Sex	0	1·1	1·2	0·8
Tumour site	9·4	2·6	4·8	5·6
Response to neoadjuvant therapy	0	0	0	0
ypT category	11·2	9·2	7·4	9·2
No. of positive LNs	3·6	30·8	40·9	25·7
Total no. of LNs examined	0·4	16·7	7·0	8·0
Lymphovascular invasion	26·8	10·5	13·6	16·9
Completeness of resection (R0/R1)	15·9	5·2	6·1	8·9
Tumour grade	7·0	3·2	2·1	4·0
Neoadjuvant treatment (NACT/NACRT)	25·4	2·5	6·8	11·4

LN, lymph node; NACT, neoadjuvant chemotherapy; NACRT, neoadjuvant chemoradiotherapy.

It is important to restate that relationships between the variables and outcome are non‐linear and their importance varies considerably according to other variables owing to higher‐order interactions. As an example, even though lymph node status was found to be the most influential marker overall, combinations of other variables would make other variables most important in individual patients. To illustrate this and demonstrate how variables interact, three example patients were considered (*Tables* 4 and 5). The technique used measures the change in prediction from the mean prediction (27·4 per cent) that can be attributed to each predictor variable. This approach (calculation of Shapley value) originates from cooperative game theory.

**Table 4:** Examples of patients at low, medium and high risk of early recurrence

	AJCC stage	Description
Low risk	Stage I: ypT0 N0 M0	A 50‐year‐old man with a GOJ adenocarcinoma who undergoes neoadjuvant chemoradiotherapy. Postoperative pathology shows ypT0 tumour (responder) with no lymphovascular invasion, R0 resection and a well differentiated tumour. None of 30 lymph nodes sampled is positive.
Medium risk	Stage II: ypT3 N0 M0	A 66‐year‐old man with an oesophageal adenocarcinoma who undergoes neoadjuvant chemoradiotherapy. Postoperative pathology shows ypT3 tumour (non‐responder), lymphovascular invasion, R0 resection and a moderately differentiated tumour. None of 30 lymph nodes sampled is positive.
High risk	Stage IIIb: ypT3 N2 M0	A 70‐year‐old woman with an oesophageal adenocarcinoma who undergoes neoadjuvant chemotherapy. Postoperative pathology shows ypT3 tumour (non‐responder), lymphovascular invasion, R1 resection and poor differentiation. Five of 30 lymph nodes sampled are positive.

GOJ, gastro‐oesophageal junction.

**Table 5:** Patient examples using final model

	%		
	Low risk	Medium risk	High risk
Baseline prediction	27·4	27·4	27·4
Age	–0·8	–0·1	+ 4·3
Sex	–0·1	–0·4	–2·0
Tumour site	–1·4	+ 9·8	+ 8·1
Response to neoadjuvant therapy	–0·5	+ 0·4	+ 0·1
ypT category	–6·5	+ 4·9	+ 3·2
No. of positive LNs	–10·0	–32·2	+ 9·7
Total no. of LNs examined	–1·2	–1·9	–3·1
Lymphovascular invasion	–7·1	+ 21·1	+ 14·7
Completeness of resection (R0/R1)	–2·3	–6·0	+ 6·1
Tumour grade	–3·0	–6·9	+ 3·6
Neoadjuvant treatment (NACT/NACRT)	+ 5·8	+ 22·2	–3·9
Final prediction	0·3	38·3	68·2

The percentage contribution of each variable in each example patient is shown. This is represented as an absolute percentage change from the mean predicted value of 27·4 per cent. A calculator for this is packaged with the online model. LN, lymph node; NACT, neoadjuvant chemotherapy; NACRT, neoadjuvant chemoradiotherapy.

## Discussion

An easy‐to‐use and robust clinical model for predicting the risk of early recurrence after surgery for oesophageal adenocarcinoma was derived in this study. It uses routinely collected clinical and pathological data that should be available for every patient; together, these allow considerably more precision in risk estimation than would be possible using individual variables that are known to be influential, such as pathological lymph node involvement. The final model demonstrated excellent discrimination, and validation techniques supported the generalizability of the approach.

In addition to prognostication, this model may be useful for planning adjuvant therapy. Early recurrence after oesophagectomy, often before recovery from surgery is complete, is a devastating outcome for patients. Targeting existing and emerging treatment combinations in this patient group to prolong time to recurrence or prevent recurrence is vital, but can only happen with accurate predictions of the likelihood of relapse. The starting point for consideration of treatment escalation or novel combinations (such as immunotherapy) after surgery is the identification of patients who are at high risk of recurrence. The authors have purposefully avoided dichotomization/stratification based on outcome, and presented raw probability in preference to this. This will allow full discussions between surgeons/oncologists and patients regarding the benefits of adjuvant therapy and tailored to the individual patient's postoperative recovery and wishes. It may also allow stratification of adjuvant trials based on layered levels of risk.

This cohort exhibited an early recurrence rate of 29·1 per cent, which is similar to that in previous reports^3^, ^4^, ^5^, ^8^ where this outcome was specified explicitly. There was also an R1 resection rate of 28·4 per cent, in line with previously reported data^34^, ^35^ based on an RCP definition of CRM positivity (CRM less than 1 mm is positive). In univariable analysis, all factors expected to correlate with worse prognosis (including ypT, ypN, lymphovascular invasion, R1 resection and grade of differentiation) were significantly worse in patients who developed early recurrence. This validates the present cohort as a true representation of contemporary practice and a sensible place to begin building more complex models.

Discrimination of the different models was similar, with minimal variability in AUC values between models on validation. However, the ensemble model consistently performed the best and is a suitable choice for the final model. The decline in performance from the training set to validation, which was particularly marked for the RF and ensemble models, is a consequence of the tuning process, whereby the optimum values are chosen from a grid of thousands after repeated tests (in this case repeated 10‐fold cross‐validation). In this setting, the apparent performance of the model on the training set is overestimated and should be disregarded.

There was marked heterogeneity in variable importance between models. This is interesting, particularly in the context of the models performing so similarly overall, and supports the idea of combining them to capture different patient information. The most important variables overall were number of positive lymph nodes and lymphovascular invasion, which accounted for 42·6 per cent of performance in the final model. This is not only biologically sensible, but the subject of several recent publications^12^, ^36^, ^37^ and ongoing translational work. Although not available for this study, more detail regarding lymphadenopathy, such as downstaging and anatomical location, would probably be informative. It is difficult to reach firm conclusions regarding variables considering the nature of the study. However, the authors draw attention to two facets of the model. First, TRG was the least influential variable across the board, with an importance of almost 0 per cent. This suggests that in itself TRG adds no information over the other measured variables in predicting early outcomes. This is in keeping with emerging data regarding the genomic disparity between primary tumours and their metastasis (lymph node or distant)^38^, and a previous report^12^ of the importance of lymph node downstaging to clinical outcome. Second, type of treatment was the third most important determinant of outcome, with NACT having an advantage over NACRT. In this cohort, although the postoperative pathology was considerably more favourable after NACRT, the rate of early recurrence was no less, and tended to be higher (NACRT 35·5 per cent, NACT 27·5 per cent; *P* = 0·061 (*Table* *S2* *,* supporting information)). This suggests that, although postoperative pathology is more favourable with NACRT, this does not translate to better outcome^39^, ^40^, ^41^; hence ypT3 N1 R0 status after NACT does not have the same meaning as a ypT3 N1 R0 result after NACRT, at least in the early phase after treatment. This is important in postoperative discussions with patients. As the machine learning approaches detailed here allow interactions between variables, the model suggests that NACRT confers a greater risk; however, this increased risk is conditional on the other variables being static rather than an overall increase in risk from having NACRT.

To explore this further, details of recurrence location (locoregional *versus* distant) would be informative. However, owing to the historical nature of the data for the majority of the patients (collected for the first study) it was not possible to ascertain this reliably for most of the cohort. The concern with NACRT is that improved locoregional control is at the expense of undertreatment of microscopic distant disease, particularly where the radiotherapy field is limited anatomically (for example with GOJ tumours). The expected consequence of this would be fewer locoregional recurrences and more distant recurrences, although this has not been demonstrated in other comparative studies and a recently published RCT^41^.

The present study lacks the number of patients needed to separately analyse the influence of neoadjuvant treatment on oesophageal and GOJ tumours, however, the individual variable importance calculation available in the web app allows some insight to be gained. Here, the relative negative influence of NACRT (increased risk of recurrence compared with NACT) is, on the whole, more pronounced for GOJ tumours compared with oesophageal tumours (an example of a second‐order interaction), despite the recurrence rate being higher for oesophageal than GOJ tumours.

Other risk factors for early recurrence, including perioperative blood transfusion^42^, complications of surgery^43^ and preoperative staging, were not available for this study, but are less discriminatory. Nor were precise neoadjuvant regimens available for all patients. It is therefore unclear whether these results would be influenced by completion of treatment as prescribed, or indeed by whether any adjuvant therapy was given. The absence of these factors seems to have minimal effect on the model, suggesting a small margin of effect on outcomes. Combining these factors could potentially increase the performance of the present model if incorporated in the future. Ultimately, differential gene expression and mutation^44^, ^45^ may well determine prognostication and treatment pathways^46^, but such data are unlikely to be available universally for some years. Until then, clinical and histopathological data remain the standard.

In that context, gains from mathematical and computer‐based techniques are key to precision in delivery of cancer care. Here, several modern approaches that produce viable models were demonstrated. This study used a data set that was relatively small and simple in a machine learning context, and the improvement in performance over a standard logistic regression was small (internal validation AUC 0·781). This is nonetheless important as such an improvement is in effect ‘free’. The strengths of this study lie in its multicentre nature and the heterogeneity of the cohort. This approach should maximize the utility of the model on external populations. All the data points used should be collected routinely at the majority of institutions, which should allow uptake without change in practice. The College of American Pathologists (CAP) definition of CRM positivity (CRM positive if there is tumour at the resection margin) was derivable for centre G, and performance was preserved in this subgroup if that definition was used instead of the RCP definition (AUC 0·813 with model generated on centres A–F (650 patients) and validated on centre G (162)) (*Fig*. *S2*, supporting information), supporting utility in both settings. The study focused on predictive model study design and reporting as suggested by the AJCC^47^ and TRIPOD^48^ statements.

The training set was limited to patients undergoing neoadjuvant therapy for adenocarcinoma of the oesophagus. No attempt was made to apply the model to a chemotherapy‐naive population, and it is unlikely to calibrate well in this group owing to the differing influence on survival of yp compared with p staging^49^. It is also unclear whether the model would be valid in patients with squamous cell carcinoma; the authors advocate an early external validation exercise using this patient group. A formal prospective validation/recalibration using the CAP definition of CRM positivity would also be beneficial. Simulation studies have suggested that 100–200 cases (positives) are required for accurate validation^50^, which, assuming a stable incidence, would require approximately 380–760 patients. A further limitation was the significant proportion of the original patients with missing data, which will have introduced a degree of selection bias. Multiple imputation is possible as a means of addressing this, but was considered less appropriate in this study because of the high proportion of missing data for the outcome measure and the lack of an external validation set.

A large multicentre cohort of patients who underwent oesophagectomy has been used to derive an accurate prediction model for early cancer recurrence, with excellent performance on validation. Machine learning techniques represent an attractive proposition for maximizing performance of predictive models.

## Supporting information


**Appendix S1**. Supporting Information
