# Supplementary material for: Machine learning to predict early recurrence after oesophageal cancer surgery
Source: Br J Surg. 2020 Jan 30;107(8):1042–52. doi: 10.1002/bjs.11461 (PMC7299663; doi:10.1002/bjs.11461)
Supplement: Supplementary file 1 — Appendix S1. Supporting Information [file BJS-107-1042-s001.docx]

**BJS11461**

**Machine learning to predict early recurrence after oesophageal cancer surgery**

S. A. Rahman, R. C. Walker, M. A. Lloyd, B. L. Grace, G. I. van Boxel, B. F. Kingma, J. P. Ruurda, R. van Hillegersberg, S. Harris, S. Parsons, S. Mercer, E. A. Griffiths, J. R. O’Neill, R. Turkington, R. C. Fitzgerald and T. J. Underwood, on behalf of the OCCAMS Consortium

**Appendix S1 Oesophageal Cancer Clinical and Molecular Stratification (OCCAMS) Consortium Members List (May 2019)**

Ayesha Noorani^1^, Rachael Fels Elliott^1^, Paul A.W. Edwards^1,2^, Nicola Grehan^1^, Barbara Nutzinger^1^, Jason Crawte^1^, Hamza Chettouh^1^, Gianmarco Contino^1^, Xiaodun Li^1^, Eleanor Gregson^1^, Sebastian Zeki^1^, Rachel de la Rue^1^, Shalini Malhotra^1,3^, Simon Tavaré^2^, Andy G. Lynch^2^, Mike L. Smith^2^, Jim Davies^5^, Charles Crichton^5^, Nick Carroll^6^, Peter Safranek^6^, Andrew Hindmarsh^6^, Vijayendran Sujendran^6^, Stephen J. Hayes^7,14^, Yeng Ang^7,8,29^, Shaun R. Preston^9^, Sarah Oakes^9^, Izhar Bagwan^9^, Vicki Save^10^, Richard J.E. Skipworth^10^, Ted R. Hupp^10^, J. Robert O'Neill^10,23^, Olga Tucker^11,33^, Andrew Beggs^11,28^, Philippe Taniere^11^, Sonia Puig^11^, Timothy J. Underwood^12,13^, Fergus Noble^12^, James P. Byrne^12^, Jamie J. Kelly^12^, Jack Owsley^12^, Hugh Barr^15^, Neil Shepherd^15^, Oliver Old^15^, Jesper Lagergren^16,25^, James Gossage^16,24^, Andrew Davies^16,24,^ Fuju Chang^16,24^, Janine Zylstra^16,24^, Vicky Goh^24^, Francesca D. Ciccarelli^24^, Grant Sanders^17^, Richard Berrisford^17^, Catherine Harden^17^, David Bunting^17^, Mike Lewis^18^, Ed Cheong^18^, Bhaskar Kumar^18^, Simon L. Parsons^19^, Irshad Soomro^19^, Philip Kaye^19^, John Saunders^19^, Laurence Lovat^20^, Rehan Haidry^20^, Victor Eneh^20^, Laszlo Igali^21^, Michael Scott^22^, Shamila Sothi^26^, Sari Suortamo^26^, Suzy Lishman^27^, George B. Hanna^31^, Christopher J. Peters^31^, Anna Grabowska^32^

^1^Medical Research Council Cancer Unit, Hutchison/Medical Research Council Research Centre, University of Cambridge, Cambridge, UK

^2^Cancer Research UK Cambridge Institute, University of Cambridge, Cambridge, UK

^3^Department of Histopathology, Addenbrooke's Hospital, Cambridge, UK

^4^Oxford ComLab, University of Oxford, UK, OX1 2JD

^5^Department of Computer Science, University of Oxford, UK, OX1 3QD

^6^Cambridge University Hospitals NHS Foundation Trust, Cambridge, UK, CB2 0QQ

^7^Salford Royal NHS Foundation Trust, Salford, UK, M6 8HD

^8^Wigan and Leigh NHS Foundation Trust, Wigan, Manchester, UK, WN1 2NN

^9^Royal Surrey County Hospital NHS Foundation Trust, Guildford, UK, GU2 7XX

^10^Edinburgh Royal Infirmary, Edinburgh, UK, EH16 4SA

^11^University Hospitals Birmingham NHS Foundation Trust, Birmingham, UK, B15 2GW

^12^University Hospital Southampton NHS Foundation Trust, Southampton, UK, SO16 6YD

^13^Cancer Sciences Division, University of Southampton, Southampton, UK, SO17 1BJ

^14^Faculty of Medical and Human Sciences, University of Manchester, UK, M13 9PL

^15^Gloucester Royal Hospital, Gloucester, UK, GL1 3NN

^16^St Thomas's Hospital, London, UK, SE1 7EH

^17^Plymouth Hospitals NHS Trust, Plymouth, UK, PL6 8DH

^18^Norfolk and Norwich University Hospital NHS Foundation Trust, Norwich, UK, NR4 7UY

^19^Nottingham University Hospitals NHS Trust, Nottingham, UK, NG7 2UH

^20^University College London, London, UK, WC1E 6BT

^21^Norfolk and Waveney Cellular Pathology Network, Norwich, UK, NR4 7UY

^22^Wythenshawe Hospital, Manchester, UK, M23 9LT

^23^Edinburgh University, Edinburgh, UK, EH8 9YL

^24^King's College London, London, UK, WC2R 2LS

^25^Karolinska Institutet, Stockholm, Sweden, SE‐171 77

^26^University Hospitals Coventry and Warwickshire NHS, Trust, Coventry, UK, CV2 2DX

^27^Peterborough Hospitals NHS Trust, Peterborough City Hospital, Peterborough, UK, PE3 9GZ

^28^Institute of Cancer and Genomic sciences, University of Birmingham, B15 2TT; ^29^GI science centre, University of Manchester, UK, M13 9PL

^30^Queen's Medical Centre, University of Nottingham, Nottingham, UK, NG7 2UH

^31^Imperial College NHS Trust, Imperial College London, UK, W2 1NY

^32^Queen's Medical Centre, University of Nottingham, Nottingham, UK

^33^Heart of England NHS Foundation Trust, Birmingham, UK, B9 5SS

**Appendix S2 R-Code**

####The below code will train an elastic net, random forest, XGboost and ensemble models on data

#### provided, and internally validate them. Descriptions of how to use the model on batches of new data

#### (e.g. for validation are also provided) Saqib A Rahman August 2019 s.rahman@soton.ac.uk.

####Installs required packages

packages <- c("caret", "pROC", "gbm", "caretEnsemble", "ResourceSelection", “doParallel”)

if (length(setdiff(packages, rownames(installed.packages()))) > 0) {

install.packages(setdiff(packages, rownames(installed.packages())))

}

####Loads required packages

library(caret) ###Trains the models

library(pROC) ###Generates receiver operator characteristic curves

library(gbm) ###Generates Calibration plot

library(caretEnsemble)###Combines models

library(ResourceSelection)###Hosmer-Lemeshow test

library(doParallel)###Parallel processing

###Prepare/import data, label file as ‘data’. Include only columns that are to be included in the model.

###Place the dependent variable as the last column and label it as 'Outcome'. Must be a binary factor

###Ensure that variables are labelled correctly (i.e. ordinal/categorical/continuous)

###Returns a dataframe, 'dataC' which contains only complete cases.

dataC<-na.omit(data)

###Multiple imputation (such as in the MICE package) may be useful if there is a large amount of missing

###data, however will introduce bias over-optimism of performance in Internal validation metrics and

###should be assessed on external data (or a hold-out set) unless models can be pooled using Rubin’s rules

###This is possible for logistic/linear/cox regression using package ‘rms’, but not for ML models at present.

###Confirms levels of the dependent variable

levels(dataC$Outcome)<- c("No", "Yes")

###Returns a generic formula, 'fm' for the model

p<-Outcome

fm <- as.formula( paste( p, ".", sep=" ~ "))

###Returns rules for model training and hyperparameter tuning.

###'logloss' will result in 10 fold cross validation, repeated 5 times

###with the reported hyperparameters optimised for log-loss (which should optimise

###the probabilities of the predictions).

###'bootstrap' will perform the 0.632 bootstrap with 1000 resamples.

###If used with fixed hyperparameters then this will internally validate the model

logloss=trainControl(method="repeatedcv",

number=10,

repeats=5,

classProbs=TRUE,

savePredictions=TRUE,

summaryFunction = mnLogLoss)

bootstrap <- trainControl(method="boot632", number=1000,returnResamp = "all",

classProbs = TRUE, summaryFunction = twoClassSummary, savePredictions = TRUE)

####ELASTIC NET########

####Tunes hyperparameters and stores in 'tuningmodel'. Can fix the alpha to a set level if desired.

tuningmodel<- train(fm, data=dataC, method = "glmnet", trControl = logloss,metric = "logLoss",

tuneGrid = expand.grid(alpha = seq(0,1,by=0.1),lambda = seq(0.001,0.1,by = 0.001)))

tuningmodel

###Trains the final model.

FinalEL<-train(fm, data=dataC, method = "glmnet", trControl = bootstrap,metric = "ROC",

tuneGrid = expand.grid(alpha = tuningmodel$bestTune$alpha.,lambda = tuningmodel$bestTune$lambda))

###Returns the apparent AUC from the final EL model

getTrainPerf(FinalEL)

###Trains Random Forest Model with hyperparameters according to ‘tgrid’

tgrid<-expand.grid(

.mtry=2:10,

.splitrule=c("gini","extratrees"),

.min.node.size=c(1,3,5)

)

RFModel<-train(fm, data=dataC, method="ranger", num.trees=1000, na.action=na.pass,replace=TRUE,

trControl= logloss, tuneGrid=tgrid, metric ="ROC")

###Trains the final Random Forest model

tgrid2<-expand.grid(

.mtry=RFModel$bestTune$mtry,

.splitrule=RFModel$bestTune$splitrule,

.min.node.size=RFModel$bestTune$min.node.size

)

FinalRF<-train(fm, data=dataC, method="ranger", num.trees=1000, na.action=na.pass,replace=TRUE,

trControl= bootstrap, tuneGrid=tgrid2, metric ="ROC")

###Returns the apparent AUC from the final RF model

getTrainPerf(FinalRF)

####Trains XGB Model according to tuning parameters in ‘tune_grid’

####This modelling will take a large amount of time – hours to days. Parallel processing as below will reduce this.

cl<-makePSOCKcluster(detectCores(logical=FALSE)-1)

registerDoParallel(cl)

tune_grid <- expand.grid(

nrounds = seq(from = 100, to = 10000, by = 100),

eta = c(0.025, 0.05, 0.1, 0.3),

max_depth = c(2, 3, 4, 5, 6),

gamma = c(0,0.05,0.1,0.5,0.7,0.9,1.0),

colsample_bytree = c(0.4,0.6,0.8,1.0),

min_child_weight = c(1,2,3),

subsample = c(0.5,0.75,1.0)

)

xgb_tune <-train(fm,

data=dataC,

method="xgbTree",

trControl=logloss,

tuneGrid=tune_grid,

verbose=T,

metric="ROC")

####Returns Final XGB model

final_grid <- expand.grid(

nrounds = xgb_tune$bestTune$nrounds,

eta = xgb_tune$bestTune$eta,

max_depth = xgb_tune$bestTune$max_depth,

gamma = xgb_tune$bestTune$gamma,

colsample_bytree = xgb_tune$bestTune$colsample_bytree,

min_child_weight = xgb_tune$bestTune$min_child_weight,

subsample = xgb_tune$bestTune$subsample

)

FinalXGB <-train(fm,

data=dataC,

method="xgbTree",

trControl=bootstrap,

tuneGrid=final_grid,

verbose=T,

metric="ROC")

###Stops parallel processing

stopCluster(cl)

registerDoSEQ()

###Returns the apparent AUC from the final XGB model

getTrainPerf(FinalXGB)

####Ensembles the Models into one model using linear blend

EnsList<-caretList(fm,data=dataC, trControl=logloss,

tuneList=list(

ranger=caretModelSpec(method="ranger", num.trees=1000,tuneGrid=tgrid2, importance="impurity")),

glmnet=caretModelSpec(method="glmnet",tuneGrid = expand.grid(alpha = FinalEL$bestTune$alpha,lambda = FinalEL$bestTune$lambda)),

xgbTree=caretModelSpec(method="xgbTree", tuneGrid=final_grid)

))

FinalEns<-caretEnsemble(

EnsList,

metric="ROC",

trControl=trainControl(

method="repeatedCV",

number=10,

repeats=5,

classProbs=TRUE,

savePredictions=TRUE,

summaryFunction = mnLogLoss,

verbose=TRUE

))

###Returns the apparent ROC and Calibration Chart

X<-predict(FinalEL, type="prob")

X1<-X[,2]

X2<-as.numeric(dataC[,o])-1

X3<-cbind2(X1,X2)

XR<-predict(FinalRF, type="prob")

X1R<-XR[,2]

X2R<-as.numeric(dataC[,o])-1

X3R<-cbind2(X1R,X2R)

XX<-predict(FinalXGB, type="prob")

X1X<-XX[,2]

X2X<-as.numeric(dataC[,o])-1

X3X<-cbind2(X1X,X2X)

XE<-predict(FinalEns,type="prob")

X1E<-XE

X2E<-as.numeric(dataC[,o])-1

X3E<-cbind2(X1E,X2E)

par(mfrow=c(2,2))

FinalModelROC <- plot.roc(X3[,2], X3[,1],

main="Elastic Net",

grid=TRUE, auc=TRUE,print.auc=TRUE, percent=TRUE,

xlim=c(100, 0), ylim=c(0, 100),axis(1, at=c(100,0)),

xlab="Specificity (%)", ylab="Sensitivity (%)",

ci=TRUE)

ciroccurve <- ci.se(FinalModelROC,specificities = seq(0, 100, 5), boot.n=1000)

plot(ciroccurve, type = "shape", col = "lightgrey")

RFModelROC <- plot.roc(X3R[,2], X3R[,1],

main="RandomForest",

grid=TRUE, auc=TRUE,print.auc=TRUE, percent=TRUE,

xlim=c(100, 0), ylim=c(0, 100),axis(1, at=c(100,0)),

xlab="Specificity (%)", ylab="Sensitivity (%)",

ci=TRUE)

ciroccurveR <- ci.se(RFModelROC,specificities = seq(0, 100, 5), boot.n=1000)

plot(ciroccurveR, type = "shape", col = "lightgrey")

XGBModelROC <- plot.roc(X3X[,2], X3X[,1],

main="XG Boost",

grid=TRUE, auc=TRUE,print.auc=TRUE, percent=TRUE,

xlim=c(100, 0), ylim=c(0, 100),axis(1, at=c(100,0)),

xlab="Specificity (%)", ylab="Sensitivity (%)",

ci=TRUE)

ciroccurveX <- ci.se(XGBModelROC,specificities = seq(0, 100, 5), boot.n=1000)

plot(ciroccurveX, type = "shape", col = "lightgrey")

EnsModelROC <- plot.roc(X3E[,2], X3E[,1],

main="Ensemble",

grid=TRUE, auc=TRUE,print.auc=TRUE, percent=TRUE,

xlim=c(100, 0), ylim=c(0, 100),axis(1, at=c(100,0)),

xlab="Specificity (%)", ylab="Sensitivity (%)",

ci=TRUE)

ciroccurveE <- ci.se(EnsModelROC,specificities = seq(0, 100, 5), boot.n=1000)

plot(ciroccurveE, type = "shape", col = "lightgrey")

{par(mfrow=c(2,2))

calibrate.plot(X3[,2],X3[,1], ylab="Observed Probability", xlab="Predicted Probability",main="Elastic Net")

calibrate.plot(X3R[,2],X3R[,1], ylab="Observed Probability", xlab="Predicted Probability", main="Random Forest")

calibrate.plot(X3X[,2],X3X[,1], ylab="Observed Probability", xlab="Predicted Probability", main="XG Boost")

calibrate.plot(X3E[,2],X3E[,1], ylab="Observed Probability", xlab="Predicted Probability", main="Ensemble")

}

###Returns the Hosmer-Lemeshow test for the final model with 10 bins

HLFull<-hoslem.test(X3[,2],X3[,1],g=10)

HLFull

HLRFull<-hoslem.test(X3R[,2],X3R[,1],g=10)

HLRFull

HLXFull<-hoslem.test(X3X[,2],X3X[,1],g=10)

HLXFull

HLEFull<-hoslem.test(X3E[,2],X3E[,1],g=10)

HLEFull

###Internal Validation via bootstrapping - gives the final model ROC with 95%CI for 1000 bootstrap samples

###(based on the 'bootstrap' trControl function)

par(mfrow=c(1,4))

BootELROC <- plot.roc(as.numeric(FinalEL$trainingData$.outcome=='Yes'), aggregate(Yes~rowIndex,FinalEL$pred,mean)[,'Yes'],

main="Elastic Net",

grid=TRUE, auc=TRUE,print.auc=TRUE, percent=TRUE,

xlim=c(100, 0), ylim=c(0, 100),axis(1, at=c(100,0)),

xlab="Specificity (%)", ylab="Sensitivity (%)",

ci=TRUE)

BootRFROC <- plot.roc(as.numeric(FinalRF$trainingData$.outcome=='Yes'), aggregate(Yes~rowIndex,FinalRF$pred,mean)[,'Yes'],

main="Random Forest",

grid=TRUE, auc=TRUE,print.auc=TRUE, percent=TRUE,

xlim=c(100, 0), ylim=c(0, 100),axis(1, at=c(100,0)),

xlab="Specificity (%)", ylab="Sensitivity (%)",

ci=TRUE)

BootXGBROC <- plot.roc(as.numeric(FinalXGB$trainingData$.outcome=='Yes'), aggregate(Yes~rowIndex,FinalXGB$pred,mean)[,'Yes'],

main="XG Boost",

grid=TRUE, auc=TRUE,print.auc=TRUE, percent=TRUE,

xlim=c(100, 0), ylim=c(0, 100),axis(1, at=c(100,0)),

xlab="Specificity (%)", ylab="Sensitivity (%)",

ci=TRUE)

BootENS<- plot.roc(as.numeric(FinalEns$ens_model$trainingData$.outcome=='Yes'), aggregate(Yes~rowIndex,FinalEns$ens_model$pred,mean)[,'Yes'],

main="Ensemble",

grid=TRUE, auc=TRUE,print.auc=TRUE, percent=TRUE,

xlim=c(100, 0), ylim=c(0, 100),axis(1, at=c(100,0)),

xlab="Specificity (%)", ylab="Sensitivity (%)",

ci=TRUE)

###To perform internal-external validation - create datafiles with only and without

###each 'centre' to be tested. Then train models as above on each of the datasets with a centre missing,

###and use the ‘predict’ function on the dataset with only that centre to return results, example below.

Xa1<-predict(ELNa, newdata=data3, type="prob")

X1a1<-Xa1[,2]

X2a1<-as.numeric(data3$Outcome)-1

X3a1<-cbind2(X1a1,X2a1)

###Then combine the results for each centre weighted by the number of patients per centre. Note that if predicting using new data, need to enter ‘newdata=’ argument in the predict function. If using the training data, do not put any argument, or put ‘data=’ argument instead.

#####Probability Scaling using isotonic regression, used for non-probabilistic classifiers e.g. Tree based. Isotonic regression function modified and derived from https://www.analyticsvidhya.com/blog/2016/07/platt-scaling-isotonic-regression-minimize-logloss-error/

fit.isoreg <- function(iso, x0)

{

o = iso$o

if (is.null(o))

o = 1:length(x)

x = iso$x[o]

y = iso$yf

ind = cut(x0, breaks = x, labels = FALSE, include.lowest = TRUE)

min.x <- min(x)

max.x <- max(x)

adjusted.knots <- iso$iKnots[c(1, which(iso$yf[iso$iKnots] > 0))]

fits = sapply(seq(along = x0), function(i) {

j = ind[i]

if (is.na(j)) {

if (x0[i] > max.x) j <- length(x)

else if (x0[i] < min.x) j <- 1

}

upper.step.n <- min(which(adjusted.knots > j))

upper.step <- adjusted.knots[upper.step.n]

lower.step <- ifelse(upper.step.n==1, 1, adjusted.knots[upper.step.n -1] )

denom <- x[upper.step] - x[lower.step]

denom <- ifelse(denom == 0, 1, denom)

val <- y[lower.step] + (y[upper.step] - y[lower.step]) * (x0[i] - x[lower.step]) / (denom)

val <- ifelse(val > 1, max.x, val)

val <- ifelse(val < 0, min.x, val)

val <- ifelse(is.na(val), max.x, val)

val

})

fits

}

Generate predictions, ideally on an external dataset (external_data) as described above using the predict function of caret. Then

XEN<-predict(FinalEns,newdata=external_data,type="prob")

X1EN<-XEN

X2EN<-as.numeric(external_data[,o])-1

########Below returns data before adjustment

X3EN<-cbind2(X1EN,X2EN)

########Below returns data after adjustment.

iso.model<-isoreg(X3EN[,1],X3EM[,2])

X1ENs<-fit.isoreg(iso.model,X1EN)

X3ENa<-cbind2(((X1ENs*0.9999999)+0.000000001),X2EN)

####Calibration can then be reassessed as above. For this method of calibration chart, must make sure that the values are not exactly 0 or 1, hence the multiplication/addition above.

#######Final Model Variable Importance

varImp<-varImp(FinalEns)

citation('caret')

citation('pROC')

citation('ResourceSelection')

citation('caretEnsemble')

citation(‘gbm’)

citation(‘doParallel’)

#### End

#####To use the models provided at https://uoscancer.shinyapps.io/EROC/

#####**Prepare test data to match model**;

#####Column names (case sensitive): Gender, Age, Site, NAResponder, pT, VascInv, R0, Grade, NPosLN, Nodes_Examined, EarlyRec.

#####NAResponder should be TRG 1-2 or TRG3-5 and Grade should be Good, Moderate, Poor/Anaplastic

#####Set Gender, Site, NAResponder, VascInv, R0, EarlyRec to factors and Age, pT, Grade, NPosLN and Nodes_Examined to numeric

#####Use mapvalues (case sensitive) so that gender is “Male”/”Female”, Site is “GOJ”/”Oesophagus”, NAResponder is “TRG1-2”/”TRG3-5”, VascInv is “No”/”Yes”, R0 is “R0”/”R1”, EarlyRec is “No”/”Yes”

#####Load model and isotonic model into workspace

####Run isotonic fit function (fit.isoreg, as above)

####Obtain predictions using the model calibrated using isotonic regression

XE<-predict(Ensemble, newdata=data, type="prob")

X1E<-1-XE

X1Ea<-(fit.isoreg(iso.model,X1E)*0.99999+0.0000000001)

X2E<-as.numeric(data$EarlyRec)-1

X3E<-cbind2(X1Ea,X2E)

####Use predictions as above

**Table S1 Clinicopathological information by centre**

**Table S2 NACT and NACRT characteristics**

**Fig. S1 Model Calibration**

**Table S3** **Calibration of Final model before and after adjustment**

**Fig. S2 Model Discrimination on Centre G Cohort using CAP criteria for CRM**

**Fig. S3 Internal–external validation ROC curves of final model**
